# Liquid-liquid phase separation of freely falling undercooled ternary Fe-Cu-Sn alloy

**DOI:** 10.1038/srep16335

**Published:** 2015-11-10

**Authors:** W .L. Wang, Y. H. Wu, L. H. Li, W. Zhai, X. M. Zhang, B. Wei

**Affiliations:** 1 Department of Applied Physics, Northwestern Polytechnical University, Xi’an 710072, P.R.China

## Abstract

The active modulation and control of the liquid phase separation for high-temperature metallic systems are still challenging the development of advanced immiscible alloys. Here we present an attempt to manipulate the dynamic process of liquid-liquid phase separation for ternary Fe_47.5_Cu_47.5_Sn_5_ alloy. It was firstly dispersed into numerous droplets with 66 ~ 810 μm diameters and then highly undercooled and rapidly solidified under the containerless microgravity condition inside drop tube. 3-D phase field simulation was performed to explore the kinetic evolution of liquid phase separation. Through regulating the combined effects of undercooling level, phase separation time and Marangoni migration, three types of separation patterns were yielded: monotectic cell, core shell and dispersive structures. The two-layer core-shell morphology proved to be the most stable separation configuration owing to its lowest chemical potential. Whereas the monotectic cell and dispersive microstructures were both thermodynamically metastable transition states because of their highly active energy. The Sn solute partition profiles of Fe-rich core and Cu-rich shell in core-shell structures varied only slightly with cooling rate.

Phase separation takes place in numerous categories of materials including inorganic substances^1^,^2^,^3^,^4^, complex polymers^5^,^6^ and metallic alloys^7^,^8^. It is not only a fascinating phase transition process for physical and chemical research, but also becomes an efficient approach to synthesize or prepare various advanced materials^2^,^3^,^8^. In particular, the core-shell nanostructures^9^,^10^,^11^,^12^,^13^ and uniformly dispersed microstructures^14^,^15^ may be expected from the results of appropriately controlled phase separation process. As for metallic alloys, the desirable modulation of phase separation is relatively difficult to accomplish because of the influences from gravity-driven Stokes motion under terrestrial condition. The liquid phase separation of immiscible alloys usually leads to serious macrosegregation and layered structures^16^. Although space experiments open an access to microgravity state for materials science^17^,^18^, the nongravity-relevant Marangoni convection^19^,^20^,^21^,^22^ even excludes the possibility to yield homogeneous dispersion alloys during space solidification.

From the methodology point of view, an effective route to manipulate the liquid phase separation of metallic alloys depends on the combined effects of undercooling extent, gravity level, container state and cooling rate. A certain extent of undercooling is the prerequisite to initiate the liquid phase separation and subsequently to activate the rapid solidification of immiscible alloys^23^,^24^,^25^,^26^,^27^,^28^. The container state determines the wetting environment for alloy melts and hence dominates heterogeneous nucleation and surface convection. Since both phase separation and following solidification processes rely on atomic diffusion, the cooling rate of alloy melts also serves as a controlling factor to modulate process kinetics. Meanwhile, phase-field modeling method^29^,^30^,^31^,^32^ provides another way to disclose the kinetic features of liquid phase separation in compensation for the nontransparency of metallic alloys. So far there have been rather extensive investigations on the phase separation and solidification kinetics of binary alloys, but much work remains to be done for the more complicated case of ternary alloys. Here we have chosen ternary Fe-Cu-Sn peritectic alloys as a model system, which has a large positive mixing enthalpy and thus exhibits a metastable immiscibility gap. For the specific Fe_47.5_Cu_47.5_Sn_5_ alloy, the critical undercooling of metastable liquid phase separation is only 51 K^33^,^34^. The objective of the present work is to realize the active modulation of liquid phase separation of ternary Fe_47.5_Cu_47.5_Sn_5_ alloy by containerless rapid solidification inside drop tube. When alloy droplets with diameters of several tens to hundreds micrometers are freely falling in protecting gas environment, they benefit from the combined advantages of high undercooling, emulated microgravity, containerless state and rapid cooling. Besides, phase-field simulation helps to reveal the dynamic mechanisms of liquid phase separation.

Among the three binary systems involved in ternary Fe-Cu-Sn alloys, Fe-Cu alloys represent a binary peritectic system with broad metastable immiscibility gap. Binary Fe-Sn system forms typical monotectic alloys possessing large immiscible temperature interval, while binary Cu-Sn alloys display a stable peritectic system. Ternary Fe_47.5_Cu_47.5_Sn_5_ alloy was prepared with arc melting method by adding 5 at.% Sn element into binary Fe_50_Cu_50_ alloy. DSC thermal analysis was performed at a scan rate of 10 K/min to reveal the liquid phase separation and subsequent solidification sequence. A drop tube technique with 3 m height backfilled by mixed helium and argon gases was applied to explore the metastable phase separation and rapid solidification mechanism. The macrosegregation formation, microstructural evolution and solute trapping effect are investigated in details.

Liquid ternary Fe_47.5_Cu_47.5_Sn_5_ alloy was rapidly solidified in a 3 m drop tube. The alloy was prepared from high purity elements of 99.99%Fe, 99.99%Cu and 99.999%Sn in a high vacuum arc-melting furnace. Each sample had the mass of 2 g and was placed in a 13 mm ID×15 mm OD ×160 mm quartz tube, which had a small orifice about 0.3 mm in diameter at the bottom and was installed on the top of drop tube. The drop tube was then evacuated to 2 × 10^−4^ Pa and backfilled with a gas mixture 4:1 of He (99.995%) and Ar (99.999%) to 10^5^ Pa. Superheating to about 200 K above the liquidus temperature was accomplished by RF induction heating. After that, the bulk sample was dispersed into small droplets by high-pressure Ar jetting gas, which fell down freely. The finally solidified samples were sectioned, polished and etched with a solution of 200 g CrO_3_ + 17 ml HCl + 20 g Na_2_S_2_O_3_ + 1000 ml H_2_O. Their phase constitutions were analyzed by Rigaku D/max 2500 X-ray diffractometer, the solidification microstructures and solute distribution profiles were investigated with FEI Sirion electron microscope and INCA Energy 300 energy dispersive spectrometer. The phase field method was applied to simulate the process of liquid phase separation. In addition, the liquidus phase transition temperature of Fe_47.5_Cu_47.5_Sn_5_ alloy was determined by SDT Q600 differential scanning calorimeter.

## Structure patterns of phase separation

Both DSC thermal analysis and bulk undercooling experiments^33^,^34^ indicate that liquid Fe_47.5_Cu_47.5_Sn_5_ ternary alloy does not exhibit phase separation if its undercooling is smaller than 51 K. In such a situation, it solidifies in the normal way of stable peritectic alloy. However, liquid-liquid phase separation is initiated as soon as alloy undercooling exceeds the threshold value of 51 K. At the substantially undercooled state, this alloy displays the second critical undercooling of 196 K, below which liquid phase separation proceeds only to a microscopic extent so that it still behaves much like a normal peritectic alloy. Once undercooling increases beyond 196 K, macroscopic liquid phase separation takes place before the occurrence of solid phase nucleation. Afterwards the solidification process of highly undercooled alloy melts involves three stages: firstly γFe phase nucleates and grows, subsequently the peritectic reaction L+γFe → (Cu) occurs at temperatures below 1068 K, and finally all the residual liquid phase is consumed up by another peritectic reaction L + (Cu) → β-Cu_5.6_Sn when its temperature becomes lower than 1013 K. Considering γFe phase is subject to a polymorphic transition in the due course, the phase constitution of rapidly solidified ternary Fe_47.5_Cu_47.5_Sn_5_ alloy consists of αFe and (Cu) solid solution phases together with some amount of β-Cu_5.6_Sn intermetallic compound.

**Figure 1:**
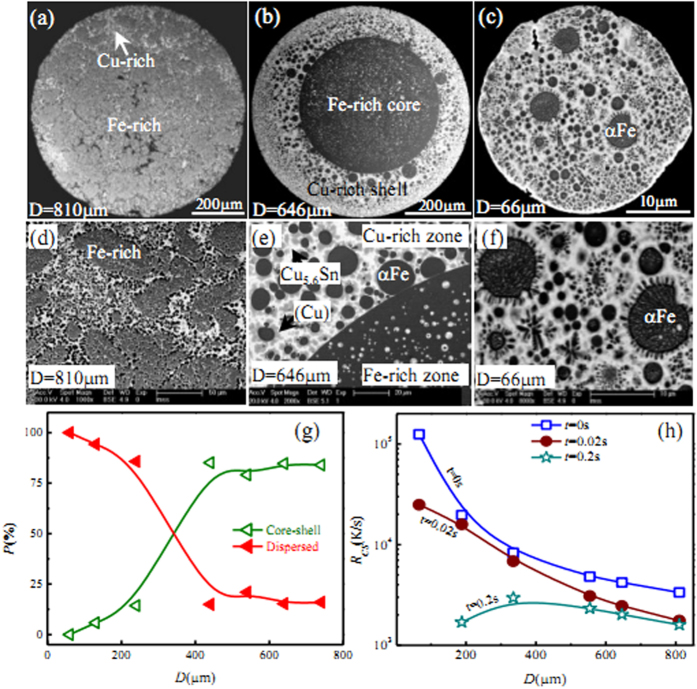
Structural morphologies induced by liquid phase separation of ternary Fe_47.5_Cu_47.5_Sn_5_ alloy versus droplet diameter. (**a–c**) macrostructures, (**d–f**) microstructures, (**g**) forming probability, and (**h**) cooling rate. The black is primary αFe phase, the grey is (Cu) phase, and the white is Cu_5.6_Sn phase respectively.

Figure 1 shows the structure patterns of ternary Fe_47.5_Cu_47.5_Sn_5_ alloy droplets under free fall condition. For the largest droplet with a diameter of 810 μm, the macrostructure displays that the nubbly Fe-rich phase forms a kind of monotectic cell microstructure which is surrounded by the Cu-rich phases, as seen in Fig. 1(a). With the decrease of droplet diameter, liquid phase separation takes place apparently and generates two-layer core-shell structure in the droplet range of 100 < D < 810 μm, where the inner part is Fe-rich core, and the outer part is Cu-rich shell, as shown in Fig. 1(b). When the droplet diameter decreases to 66μm, which is the smallest droplet during the experiments, the microstructure shows the dispersed pattern of αFe phase particles distributed into the Cu-rich matrix. In Fig. 1(c), the black is αFe phase, the grey is the (Cu) solid solution phase, and the white is Cu_5.6_Sn intermetallic compound. Therefore, the multiple solidification characteristics of liquid ternary Fe_47.5_Cu_47.5_Sn_5_ alloy appear under free fall condition: monotectic cell, core shell and dispersed structure with the decrease of droplet diameter.

The microstructural morphologies of the different alloy droplets are illustrated in Fig. 1(d-f). At the largest droplet diameter of 810 μm, primary αFe phase grows into a monotectic cell of nubbly structure, which is surrounded by the grey (Cu) solid solution phase resulting from the first peritectic reaction, that is L + γFe → (Cu). β-Cu_5.6_Sn phase is produced through the second peritectic reaction and is distributed among interdendritic gaps. Once the droplet diameter decreases from 646 to 188 μm, the Fe-rich and Cu-rich zones become clearly separated from each other and their boundary has evolved into a smooth interface, where αFe and (Cu) solid solute phases distributed into the Cu-rich and Fe-rich zones, respectively, as seen in Fig. 1(e), whereas the peritectic β-Cu_5.6_Sn phase forms around the (Cu) phase in the due sequence. As the droplet diameter reduces to 66 μm, the primary γFe phase grows in two morphologies: the equiaxed grains and the dendrite structures, which are dispersed randomly into the Cu-rich matrix, as shown in Fig. 1(f).

Based on the experimental results, the dispersed and core-shell morphologies are the main structures of ternary Fe_47.5_Cu_47.5_Sn_5_ alloy. Their forming probabilities at the different droplet diameters provide some important information to investigate the liquid phase separation characteristics of ternary Fe_47.5_Cu_47.5_Sn_5_ alloy under the free fall condition. The statistical analysis displays that the core-shell structures are most frequently generated at the intermediate droplet diameters of 200 < D ≤ 800 m, and the forming probability attains 99.1% at D = 800 μm. Whereas the dispersed structures form when the droplet diameters D < 200 μm, and the forming probability is 100% at 66 μm diameter, which is shown in Fig. 1(g). Clearly, the middle sized droplets are easy to experience macroscopic phase separation and form the core-shell structures.

## Solute concentration field and Chemical potential evolutional characteristics

Phase separation plays an important role in the final structure morphology of monotectic or peritectic alloy. The phase field method is an effective way to simulate such a complicated process. The macrostructures demonstrated in Fig. 1 suggest that the Sn solute should have stronger affinity with the Cu solvent, rather than Fe solvent. EDS analysis also reveals that Fe-rich zone contains only 1.49 at.% Sn, and Cu-rich zone dissolves about 8.25 at.% Sn. Therefore, ternary Fe_47.5_Cu_47.5_Sn_5_ alloy can be approximately regarded as the pseudo binary Fe_47.5_(Cu_0.905_Sn_0.095_)_52.5_ alloy during the phase field simulation^36^,^37^. The free energy expression is written as:


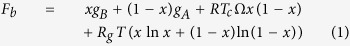


where *x* is the Fe molar mass, (1−*x*) is the Cu_0.905_Sn_0.095_ molar mass, *g*_*B*_ and *g*_*A*_ are molar free energy of Fe and Cu_0.905_Sn_0.095_ respectively, *T*_*c*_ is the critical temperature, *Ω* is the interaction parameter of alloy. The chemical potential of the alloy is expressed as:


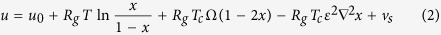


here *u*_*0*_ *=* *g*_*B*_ *−* *g*_*A*_. Based on the modified Model H, the phase field governing equation is expressed as:


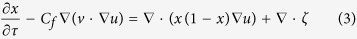



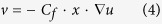



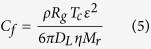


*C*_*f*_ is the fluidity parameter of alloy melt. In small droplets, the Reynolds number is less than the magnitude of 10^−3^, thus the local velocity v can be taken as *f* = −∇*F*. *ρ* is the density of liquid alloy, *R*_*g*_ the gas constant, *ε* the length scale, *D*_*L*_ the diffusion coefficient, *η* the viscosity, and *M* the molar mass.

The governing equations are dealt with a two-dimensional square grid by an explicit finite difference technique so as to simplify the numerical analysis process. This is justified by the normally spherical symmetry of the temperature and concentration fields within a freely falling alloy droplet. During the simulation, the initial velocity is zero. The liquidus temperature is 1722 K. The grid size is set as 200 × 200, the step of space is set as Δ*x* = Δ*y* = 1. The alloy droplet diameter is 200 μm. The time step Δ*τ* is 0.001 which ensures the stability of numerical solution. The surface parameters are *H* = 0.38 and *g* = 0.4. The characteristic length of spatial heterogeneity *ε* = 1.0 μm. Finally the calculations were performed in a Lenovo 1800 cluster system.

The Fe-rich and Cu-rich dispersed globules of ternary Fe_47.5_Cu_47.5_Sn_5_ alloy move mainly by Marangoni migration and Stokes motion during liquid phase separation. The Marangoni migration of second phase globule is much more complicated in comparison with the Stokes motion, which involves both thermal Marangoni migration and solutal Marangoni migration. Therefore, it is necessary to compare the influences from two kinds of Marangoni migrations with that of the Stokes motion. The Stokes motion velocity *V*_*s*_ of a single globule with radius *r* in the matrix phase is written by^35^,^36^:


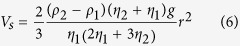


The thermal Marangoni migration velocity *V*_*mt*_ of a single globule is expressed as^36^,^38^:


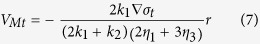


where *ρ*_1_ and *ρ*_2_ are the densities of the matrix and dispersive phase, *g* is the residual gravitational acceleration, *k*_1_ and *k*_2_ are the thermal conductivities of the matrix and dispersive phases, while *η*_1_ and *η*_2_ are their viscosities respectively, g is estimated as 10^3^ *g*_0_ in the present experiment. Here *g*_0_ = 9.8 m·s^−2^ is the normal gravitational acceleration.The interfacial tension gradient caused by temperature field is:


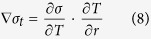


The solutal Marangoni migration velocity *V*_*Mc*_ of a single globule can be expressed by^36^,^39^:


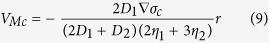


where *D*_*1*_ and *D*_*2*_ are the solute diffusion coefficients of the matrix and dispersive phases. ∇*σ*_*c*_ is the interfacial tension gradient resulting from concentration field, which is written as the following equation:


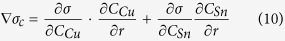


*C*_*Cu*_ and *C*_*S*n_ denote the concentration of a solute Cu and Sn, respectively. Clearly, ∂*σ*/∂*C*_*Cu*_ and ∂*σ*/∂*C*_*Sn*_ are the concentration-dependent coefficients of interfacial tension, whereas ∂*C*_*Cu*_/∂*r* and ∂*C*_*Sn*_/∂*r* are the corresponding concentration gradients.

The interfacial tension of Fe-rich and Cu-rich liquid phase is estimated on basis of Cahn-Hilliard model^36^,^40^:


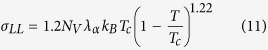


here *N*_*V*_ is the atom number of unit volume, *λ*_*α*_ the interface atom distance, *k*_*B*_ the Boltzmann constant, and *T*_*c*_ the critical temperature.

The heat transfer equation is given in polar coordinates:


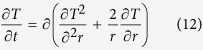


The initial and boundary conditions during free fall are given as:


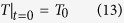



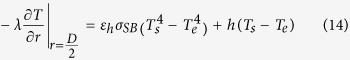


where *α* is the thermal diffusivity of alloy melt, *λ* the heat conductivity, *ε*_*h*_ the emissivity, *σ*_*SB*_ the Stefan- Boltzmann constant, *h* the heat transfer coefficient, *T*_*0*_ the initial temperature of alloy melt, *T*_*s*_ the droplet surface temperature, and *T*_*e*_ the ambient temperature.

**Figure 2:**
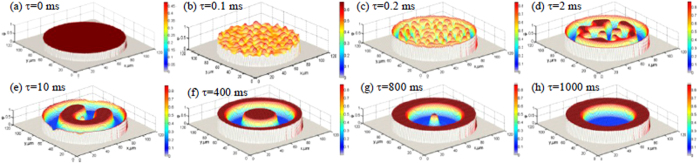
3D snapshots of solute concentration field during liquid phase separation: (**a–h**) correspond to the different moments, τ = 0, 0.1, 0.2, 2, 10, 400, 800, and 1000.

The 3D phase separation snapshots of phase field simulation for the pseudo binary Fe_47.5_(Cu_0.905_Sn_0.095_)_52.5_ alloy at different moments are presented in Fig. 2. Before the phase separation, the concentration of liquid phase remains homogeneous, which is seen in Fig. 2(a). Once the phase separation takes place, the morphology varies with the evolution time of phase separation, a large number of Fe-rich globules separate from the liquid phase and form the monotectic cell structure, which is shown in Fig. 2(b). Then the surface segregation layer forms owing to the effect of surface segregated potential. At τ = 0.2 ms, the surface segregation occurs prior to bulk decomposition, and forms the Cu-rich surface layer followed by the Fe-rich layer in the liquid phase due to the hydrodynamic and Marangoni migration. In the process of phase separation, the interfacial tension gradients drive the thermal Marangoni migration inwards and the concentration gradients tend to drive the solutal Marangoni migration outwards. With the extension of the evolution time, the Cu-rich surface segregated layer thickens gradually and the inner Cu-rich phase grows in the dispersive way. When the time extents to 0.6 ms, the second Cu-rich layer forms inside the liquid phase. The reason may be that the solutal Marangoni migration velocity is faster than the thermal Marangoni migration velocity, which leads to the Cu-rich globules collide randomly in the process of migration. When the evolution time exceeds 1 ms, the flow field quickly responses to the local force field, the inner Cu-rich phase aggregation becomes much faster through absorbing small globules around itself in the effect of the Ostwald ripening, and forms the triple-layer structure at τ = 400 ms. The surface segregated layer grows thicker and thicker by absorbing inner Cu-rich liquid phase in the following evolution, and displays the two-layer structure in the end. Obviously, the shorter evolution time results in the dispersed structure, as seen in Fig. 2(b). The longer evolution time, Marangoni migration and surface segregation can generate the core-shell structure. The monotectic cell structure is similar to the evolution profile at τ = 0.1 ms.

**Figure 3:**
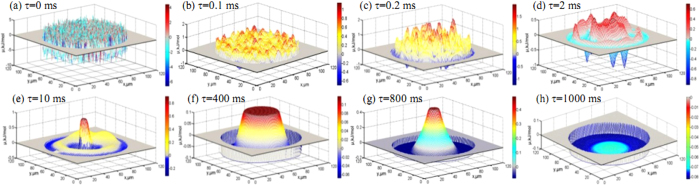
Chemical potential evolution versus liquid phase separation time: (**a–h**) correspond to the different moments, τ = 0, 0.1, 0.2, 2, 10, 400, 800, and 1000 ms.

The chemical potential reflects the system stability characteristics. Before the occurrence of phase separation, the Fe-rich and Cu-rich chemical potential distributes dispersively, which means that the alloy system is on the instable condition as the active energy is high, the maximum value is about 3.09 kJ/mol at τ = 0 ms, as shown in Fig. 3(a). With the extension of evolution time, the chemical potential plays the disorderly feature. Then the chemical potential near the droplet surface reduces quickly due to the effect of surface segregation potential, while the chemical potential at the center part shows the wave crest feature and also decreases gradually, which is illustrated in Fig. 3(b,c). Obviously, the chemical potential gradient occurs in the liquid phase. The chemical potential gradient always drives the solute to move toward the lower chemical potential on the influence of active energy, thus the surface segregation layer forms during liquid separation (τ = 400 ms). However, with the thickening of the surface segregation layer, the center chemical potential increases abruptly owing to the fact that the surface chemical potential absorbs solute from the center, which leads to the consequence that a large amount of Cu-rich solute gathers quickly, and forms the mountain-like profiles, as shown in Fig. 3(e). When the evolution time τ = 1000 ms, the hole-like feature occurs and the center chemical potential exhibits the minimum where the energy difference between the inner and outer layer is only about −0.015 kJ/mol. It is certified that the evolution system achieves the stable state here. According to the above analysis, the two-layer core-shell structure is the most stable structure in the process of liquid phase separation.

## Marangoni migration during liquid phase separation

**Figure 4:**
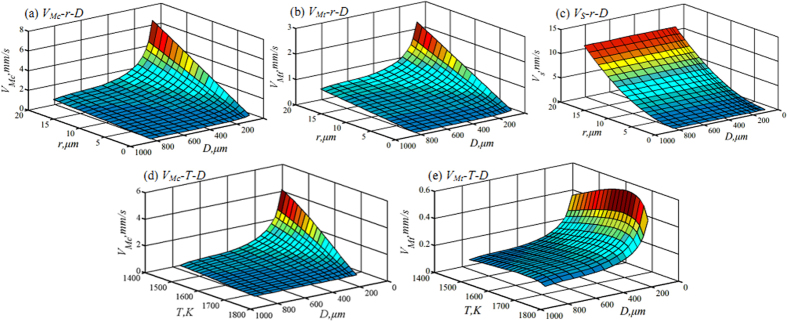
Marangoni and Stokes motion velocity at 1622 K versus globule radius, droplet diameter and temperature: (**a**) solutal Marangoni migration at 1622 K, (**b**) thermal Marangoni migration at 1622 K, and (**c**) Stokes motion velocity, (**d**) solutal Marangoni migration of 5 μm radius globule, and (**e**) thermal Marangoni migration of 5 μm radius globule.

The Marangoni migration has a significant influence on the movement of Cu-rich liquid phase during liquid phase separation, and its velocity decides the final macrostructure morphologies. Three types of motion characteristics at different droplet diameters are demonstrated in Fig. 4(a–c). Apparently, the larger globules have the larger Marangoni migration velocity for every droplet. The Stokes motion velocity of a 20 μm radius globule is only about 10.7 nm/s, where the droplet diameters vary from 810 μm to 66 μm. This is because the gravitational acceleration has been reduced to 10^−3^ g_0_ in the drop tube experiment. Stokes motion velocity is far less than the thermal and solutal Marangoni migration velocity, therefore it can be neglected under free fall condition, as shown in Fig. 4(c).

The globule radius and droplet diameter have the significant fluences on the Marangoni migration velocity. From Fig. 4(a), the Cu-rich globule with a radius of 1 μm shows a solutal Marangoni migration velocity of 26 nm/s within the largest alloy droplet of 810 μm. This increases up to 528 nm/s at the globule radius 20 μm. In contrast, it migrates at a velocity of 324 nm/s with 1 μm globule radius inside the smallest alloy droplet of 66 μm. The largest solutal Marangoni migration velocity appears at the globule radius 20 μm in this droplet, which attains 6.48 mm/s. Clearly, the solutal Marangoni migration velocity of 20 μm globule radius is almost 20 times larger than that of 1 μm globule radius. Furthermore, such a velocity for a 20 μm Cu-rich globule inside the smallest alloy droplet of 66 μm diameter is enhanced by a factor of 12 times by comparison with the droplet diameter 810 μm. Similarly, the thermal Marangoni migration velocity displays the same tendency as the solutal Marangoni migration velocity, as seen in Fig. 4(b). For example, the thermal Marangoni migration velocity of 1 μm globule achieves 21 nm/s at the 810 μm droplet diameter. It shows 421 nm/s migration velocity at the 20 μm globule radius. In the case of the smallest alloy droplet with 66 μm diameter, the thermal Marangoni migration velocity is 114 nm/s for 1 μm globule radius, whereas it amounts up to 2.27 mm/s velocity for 20 μm globule radius. The thermal Marangoni migration velocity of 66 μm droplet is 5 times as large as that in the 810 μm alloy droplet, where the globule radius is 20 μm. It is apparent that the larger globule radius and smaller droplet diameter have the faster solutal and thermal Marangoni migration velocities for ternary Fe_47.5_Cu_47.5_Sn_5_ alloy.

On the other hand, the solutal Marangoni migration is more rapid than the thermal Marangoni migration of Cu-rich globules inside Fe_47.5_Cu_47.5_Sn_5_ alloy droplets. In the case of the largest alloy droplet with 810 μm diameter, the solutal Marangoni migration velocity amounts to 528 nm/s at 20 μm globule radius, which is 1.3 times as large as the thermal Marangoni migration velocity. Many Cu-rich globules are influenced by the solutal Marangoni migration and move outside alloy droplet, while a small number of Cu-rich globules are driven toward to the droplet center under effect of the thermal Marangoni migration. With the decrease of droplet diameter, the globule movement velocity accelerates inside alloy droplet. The solutal Marangoni migration velocity achieves 6.48 mm/s inside the smallest alloy droplet of 66 μm diameter at 20 μm globule radius, which is enhanced by a factor of about 2.85 times by comparison with the droplet diameter 810 μm, as demonstrated in Fig. 4(a,b). It is apparent that the solutal Marangoni migration velocity increases farther than the thermal Marangoni migration velocity.

The solutal Marangoni migration velocity *V*_*Mc*_ increases continuously with the decrease of temperature, whether the droplet diameter is large or small, as seen in Fig. 4(d). When the temperature decreases from 1727 K to 1427 K, the solutal Marangoni migration velocity *V*_*Mc*_ increases from 3.8 nm/s to 369 nm/s for 5 μm Cu-rich globules at the droplet diameter 810 μm. In addition, it obtains the velocity from 4.6 mm/s to 4.53 mm/s when the droplet diameter is 66 μm. Therefore, when the alloy the droplet is smaller and its temperature is lower, the solutal Marangoni migration velocity becomes higher in this alloy. However, the thermal Marangoni migration velocity shows different characteristics as compared with the solutal Marangoni migration velocity, which is illustrated in Fig. 4(e). With the decrease of temperature, the thermal Marangoni migration increases gradually. It achieves the maximum velocity of 106 nm/s at 1642 K in comparison with 74 nm/s velocity at 1727 K. Then it reduces with the further drop of temperature until 759 nm/s at 1427 K. The smaller droplet has the larger thermal Marangoni migration velocity, and has the shorter phase separation time and the higher temperature to drive the Cu-rich globule inwards alloy droplet during liquid phase separation.

Based on the above analysis, it seems probable that the smaller droplets are easier to experience phase separation and form the core-shell structure owing to the higher solutal Marangoni migration velocity. However, both the largest and smallest alloy droplets with diameter of 810 and 66 μm do not form the core-shell structure. Therefore, the cooling rate may be another controlling factor during rapid solidification under free fall condition. Since the cooling rate is quite difficult to measure within the short falling time when a bulk alloy melt is dispersed into numerous small droplets to fall freely inside drop tube, the cooling rate is calculated by Equ.s 11–14.

Figure 1(h) shows the variation of center cooling rate with droplet diameter. Apparently, those droplets with diameters smaller than 200 μm possess very high initial cooling rates, but they also show much quicker decreasing tendency with the extension of falling time. For example, if the droplet diameter is the smallest, 66 μm, the center cooling rate is 1.25 × 10^5^ K/s. This droplet completely solidifies within only 0.13 s. Because of such a high cooling rate, the Fe-rich globules have no enough time to assemble together in the process of liquid phase separation, and finally form the dispersive structure. The decrease of cooling rate slows down as droplet diameter exceeds 335 μm. The cooling rate reduces to about 1.6 × 10^−3^ K/s when the droplet diameter is 810 μm, and the corresponding phase separation time is less than 1 ms. The liquid phase is on the instable condition as the active energy is high (as seen in Figs 2 and 3), thus the macrostructure shows the nubbly Fe-rich monotectic cell morphology which are surrounded by the Cu-rich phases.

In terms of the above analyses, the final structures are determined by the effects of the evolution time, chemical potential stability, surface segregation, cooling rate and Marangoni migration together. On the one hand, the shorter evolution time and the larger cooling rate bring about the dispersed structure. On the other hand, the surface segregation, the longer evolution time and the larger solutal Marangoni migration produce the core-shell structure during liquid phase separation. The two-layer core-shell structure is the most stable morphology because it has the lowest chemical potential.

## Actual solute distribution feature

**Figure 5:**
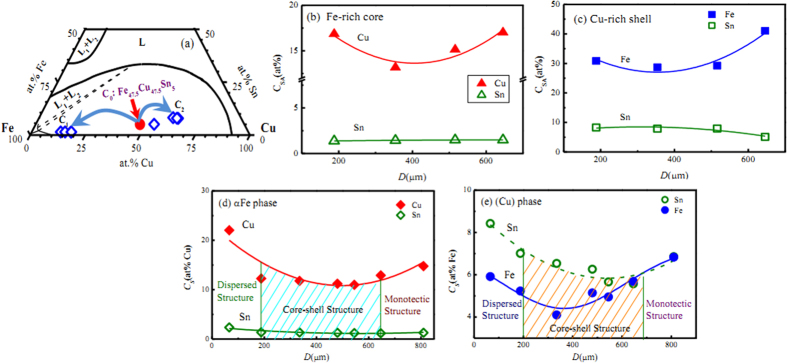
Actual solute distribution in phase separated Fe_47.5_Cu_47.5_Sn_5_ ternary alloy: (**a**) macroscopic solute separation, (**b**) average solute contents in Fe-rich core, (**c**) average solute contents in Cu-rich shell, (**d**) solute solubilities of αFe phase, (**e**) solute solubilities of (Cu) phase.

To explore the solute redistribution characteristics during liquid phase separation, the average compositions of the Fe-rich and Cu-rich zones were measured by using EDS method, which are shown in Fig. 5. The EDS analysis results demonstrate that the solutes Cu and Sn are expelled from the Fe-rich core, whereas the Fe solute is rejected from the Cu-rich shell during liquid phase separation for alloy droplets with 188~646 μm diameters. Furthermore, the macroscopic solute redistribution indicates the depletion of Sn concentration in the Fe-rich core and its enrichment in the Cu-rich shell. Figure 5(a) illustrates the average compositions of two different zones designated in the ternary Fe-Cu-Sn diagram, where the average composition of the Fe-rich core is marked as point C_1_ and the average composition of the Cu-rich shell is marked as point C_2_. Obviously, the droplet solidification process of undercooled Fe_47.5_Cu_47.5_Sn_5_ alloy involves two stages: the prior solidification of the Fe-rich core and the subsequent solidification of the Cu-rich shell. As seen in Fig. 5(b), the average Sn content of solidified Fe-rich core maintains a roughly constant value of about 1.4 at%Sn, while its average Cu content varies in the range of 13.2 ~ 17.0 at%Cu. With the decrease of droplet diameter, the average Fe content of solidified Cu-rich shell reduces from 43.7 to 28.7 at%Fe, but its average Sn content increases slightly from 4.8 to 8.3 at%Sn, which is shown in Fig. 5(c).

The actual solute distribution of primary αFe phase in Fe-rich zone and that of (Cu) phase at Cu-rich zone were measured by EDS analysis, which are illustrated in Fig. 5(d,e). Figure 5(d) shows that the Cu solubility in primary αFe phase is 14.8 at% Cu at droplet diameter D = 810 μm. It slowly decreases at first with the decrease of droplet diameter and then increases until the largest value of 22 at% Cu at D = 66 μm. A similar tendency from 6.9 to 5.9 at% Fe with decrease of droplet diameter is demonstrated in Fig. 5(e) for the Fe solubility in (Cu) phase. It is clear that the solute contents in the largest 810 μm droplet and the smallest 66 μm droplet are higher than those in other droplets displaying macrosegregation indicating that more solutes can be absorbed in the dispersed phases.

It should be noticed that the solute Sn content in the (Cu) phase is much larger than that in the primary αFe phase, indicating that the (Cu) phase has a stronger affinity with the solute Sn, which is shown in Fig. 5(d,e). In the primary αFe phase, the maximum content of Sn exhibits a sluggish increase from 1.3 to 1.4 at% Sn when the droplet diameters reduce from 810 to 188 μm, then it rapidly increases to 2.4 at% Sn at D = 66 μm, which is much smaller than the initial concentration of 5 at% Sn. In the (Cu) phase, the solubility of Sn is 6.9 at% at D = 810 μm. Subsequently, it shows the rising tendency with the decrease of droplet diameters. When the droplet diameter decreases to 66 μm, the solubility of Sn increases to 8.4 at% Sn.

## Conclusion

In conclusion, the containerless rapid solidification inside drop tube provides an efficient access to modulate or control the liquid phase separation of high-temperature metallic alloys. As for ternary Fe_47.5_Cu_47.5_Sn_5_ alloy, there appear three different kinds of phase separation patterns: monotectic cell, core shell and dispersed structures which are formed successively with the decrease of droplet diameter. The monotectic cell microstructure results from the combined effects of the moderate undercooling in the regime of 51 ~ 196 K, the short period of phase separation time less than 0.6 ms caused by the early nucleation of primary γFe phase, and the slow Marangoni migration velocity below 528 nm/s. The 3D phase field simulation discloses that the two-layer core shell structure is the most stable phase separation pattern, since it corresponds to the state with the lowest chemical potential. Such a macroscopically separated pattern requires a substantial undercooling over 196 K, a long period of phase separation time above 10 ms, and a rapid Marangoni migration velocity close to 1mm/s. Besides, the surface segregation effect is also a driving factor to yield core-shell structure. Although those smallest alloy droplets with less than 200 μm diameters may achieve the largest undercoolings and the highest Marangoni migration velocities, their very rapid cooling rates of 10^4^ ~ 10^5^ K/s allows for too short a period of liquid phase separation time. Consequently the initial phase separation configuration is quenched and “frozen down” to form the dispersed microstructures. Owing to the reduced gravity of 10^−3^ g_0_ during free fall, the Stokes motion contributes very little to the evolution of liquid phase separation. As revealed by EDS analyses, the Sn solute partition profiles of Fe-rich core and Cu-rich shell vary only slightly with droplet diameter in core-shell structures. But the solute trapping effect becomes rather conspicuous for the dispersed microstructures.

## Additional Information

**How to cite this article**: Wang, W. L. *et al.* Liquid-liquid phase separation of freely falling undercooled ternary Fe-Cu-Sn alloy. *Sci. Rep.* **5**, 16335; doi: 10.1038/srep16335 (2015).
